# Electrohydraulic lithotripsy: a safe and effective technique to retrieve an impacted Dormia basket

**DOI:** 10.1055/a-2371-1205

**Published:** 2024-08-07

**Authors:** Sébastien Carbillet, Franck Brazier, Mathurin Fumery, Clara Yzet

**Affiliations:** 1 36673 Gastroenterology, Centre Hospitalier Universitaire Amiens-Picardie, Amiens, France

Choledocholithiasis is a common indication for endoscopic retrograde cholangiopancreatography (ERCP). Mechanical lithotripsy is a frequently used technique for the extraction of complex common bile duct (CBD) stones; however, impaction of the Dormia basket around a large stone and fracture of Dormia basket wires have been described in up to 6% of procedures 1 2 3 . This complication is one of the most challenging to treat, often requiring endoscopic, percutaneous, or surgical intervention, with a risk of morbidity and mortality.

We present the case of a 47-year-old woman who had been experiencing abdominal pain and jaundice for 10 days. A computed tomography scan revealed a dilated CBD, measuring 15 mm. An endoscopic ultrasound (EUS) identified a stone at the junction between the CBD and the cystic duct. Mechanical lithotripsy was attempted via ERCP, and the stone was grasped with the Dormia basket. Multiple attempts to crush and retrieve the stone were unsuccessful, resulting in impaction of the Dormia basket in the CBD owing to a wire fracture in the operating channel. We then decided to perform electrohydraulic lithotripsy (EHL) to dislodge the stone and retrieve the impacted basket ( Video 1 ). An intraductal cholangioscope (SpyGlass) was introduced into the CBD and placed in contact with the stone, allowing EHL to be applied to pulverize the stone and enabling the removal of the Dormia basket with a grasper. The stone fragments were then retrieved with an extraction balloon.

Electrohydraulic lithotripsy is performed to fragment an impacted common bile duct stone, allowing safe retrieval of an impacted Dormia basket.Video 1

Cholangioscopy-guided EHL is a safe and effective method for the retrieval of an impacted Dormia basket. This technique is a less invasive alternative to surgery.

Endoscopy_UCTN_Code_TTT_1AR_2AH

## Notes

Endoscopy E-Videos https://eref.thieme.de/e-videos
*E-Videos* is an open access online section of the journal *Endoscopy* , reporting on interesting cases and new techniques in gastroenterological endoscopy. All papers include a high-quality video and are published with a Creative Commons CC-BY license. Endoscopy E-Videos qualify for HINARI discounts and waivers and eligibility is automatically checked during the submission process. We grant 100% waivers to articles whose corresponding authors are based in Group A countries and 50% waivers to those who are based in Group B countries as classified by Research4Life (see: https://www.research4life.org/access/eligibility/ ). This section has its own submission website at https://mc.manuscriptcentral.com/e-videos .
